# Effects of zinc supplementation on diabetes mellitus: a systematic review and meta-analysis

**DOI:** 10.1186/1758-5996-4-13

**Published:** 2012-04-19

**Authors:** R Jayawardena, P Ranasinghe, P Galappatthy, RLDK Malkanthi, GR Constantine, P Katulanda

**Affiliations:** 1 Diabetes Research Unit, Department of Clinical Medicine, Faculty of Medicine, University of Colombo, Colombo, Sri Lanka; 2 Institute of Health and Biomedical Innovation, Queensland University of Technology, Brisbane, QLD, Australia; 3 Department of Pharmacology, Faculty of Medicine, University of Colombo, Colombo, Sri Lanka; 4 Department of Applied Nutrition, Faculty of Livestock, Fisheries and Nutrition, Wayamba University of Sri Lanka, Makandura, Sri Lanka

**Keywords:** Diabetes mellitus, Zinc Supplementation, Humans, Meta-analysis

## Abstract

The number of people with diabetes and pre-diabetes are exponentially increasing. Studies on humans have shown the beneficial effects of Zinc supplementation in patients with diabetes. The present study aims to systematically evaluate the literature and meta-analyze the effects of Zinc supplementation on diabetes. A systematic review of published studies reporting the effects of Zinc supplementations on diabetes mellitus was undertaken. The literature search was conducted in the following databases; PubMed, Web of Science and SciVerse Scopus. A meta-analysis of studies examining the effects of Zinc supplementation on clinical and biochemical parameters in patients with diabetes was performed. The total number of articles included in the present review is 25, which included 3 studies on type-1 diabetes and 22 studies on type-2 diabetes. There were 12 studies comparing the effects of Zinc supplementation on fasting blood glucose in patients with type-2 diabetes. The pooled mean difference in fasting blood glucose between Zinc supplemented and placebo groups was 18.13mg/dl (95%CI:33.85,2.41; *p*<0.05). 2-h post-prandial blood sugar also shows a similar distinct reduction in (34.87mg/dl [95%CI:75.44; 5.69]) the Zinc treated group. The reduction in HbA1c was 0.54% (95%CI:0.86;0.21) in the Zinc treated group. There were 8 studies comparing the effects of Zinc supplementation on lipid parameters in patients with type-2 diabetes. The pooled mean difference for total cholesterol between Zinc supplemented and placebo groups was 32.37mg/dl (95%CI:57.39,7.35; *p*<0.05). Low-density lipoprotein cholesterol also showed a similar distinct reduction in the Zinc treated group, the pooled mean difference from random effects analysis was 11.19mg/dl (95%CI:21.14,1.25; *p*<0.05). Studies have also shown a significant reduction in systolic and diastolic blood pressures after Zinc supplementation. This first comprehensive systematic review and meta-analysis on the effects of Zinc supplementation in patients with diabetes demonstrates that Zinc supplementation has beneficial effects on glycaemic control and promotes healthy lipid parameters. Further studies are required to identify the exact biological mechanisms responsible for these results.

## Introduction

The number of people with diabetes and pre-diabetes are exponentially increasing worldwide due to population growth, aging, urbanization, unhealthy eating habits, increasing prevalence of obesity and physical inactivity [1]. Diabetes mellitus is a leading cause of morbidity and mortality worldwide, with an estimated 346 million adults being affected in year 2011 [2]. The prevalence is expected to double between years 20052030, with the greatest increases expected in low- and middle-income developing countries of the African, Asian, and South American regions [2,3]. At present, 80% of the worlds population with diabetes live in low- and middle-income countries [2,4]. Diabetes is also associated with a host of life threatening and potentially disabling macro- and micro-vascular complications [5]. Hence, there is also a much larger burden in the form of lost productivity as a result of restricted daily activity.

Ninety percent of those with diabetes have type-2 diabetes, characterized by insulin resistance, hyper insulinaemia, -cell dysfunction and subsequent cell failure [6]. Insulin, is stored as a hexamer containing two Zinc ions in -cells of the pancreas and released into the portal venous system at the time of -cells de-granulation [7]. The Zn(II) ions which are co-secreted with insulin suppress inherent amyloidogenic properties of monomeric insulin [8]. Zalewski, et al. showed that high concentrations of glucose and other secretagogues decrease the islet cell labile Zinc and video fluorescence analysis showed Zinc concentrated in the islet cells was related to the synthesis, storage and secretion of insulin [9]. *In vitro* data suggests that insulin binds to isolated liver membranes to a greater extent and that there is less degradation when co-administered with Zinc [10]. Zinc is important in insulin action and carbohydrate metabolism [11]. Oxidative stress plays an important role in the pathogenesis of diabetes and its complications. Zinc is a structural part of key anti-oxidant enzymes such as superoxide dismutase, and Zinc deficiency impairs their synthesis, leading to increased oxidative stress [12]. Studies have shown that diabetes is accompanied by hypozincemia [13] and hyperzincuria [14]. In addition Zinc deficiency is more common in developing countries [15], where diabetes is also showing an exponential increase in prevalence [2].

Animal studies have shown that Zinc supplementation improves fasting insulin level and fasting glucose in mice [16]. Human studies have also shown the beneficial effects of Zinc supplementation in both type-1 [17,18] and type-2 diabetes [19,20]. However, results of isolated randomized controlled trials are frequently contradicted by subsequent studies [21]. Especially, in type-1 diabetes studies have reported a negative effect of Zinc supplementation on glucose homeostasis [22]. Even under the most rigorous study design conditions, a well-planned single study, rarely provides definitive results and changing clinical practices relying on a single high-profile clinical trial can be harmful to patients health [23]. Well-designed randomized controlled trials are excellent when looking at effectiveness, though many fall short in reporting of safety and adverse events associated with an intervention. Systematic reviews often have increased power and decreased bias as compared with the individual studies they include, and the careful pooling of treatment effects can provide the most accurate overall assessment of an intervention [23]. Presently there are no systematic reviews exploring the therapeutic efficacy of Zinc supplementation in humans with diabetes. The study aims to systematically evaluate the literature and meta-analyze the effects of Zinc supplementation in humans with diabetes and evaluate potential toxic effects advocating against regular supplementation highlighted in the literature.

## Methods

### Literature search

A systematic review of published studies reporting the effects of Zinc supplementations on diabetes mellitus was undertaken in accordance with the Preferred Reporting Items for Systematic reviews and Meta-Analyses (PRISMA) statement for systematic reviews of interventional studies [24]. A three staged comprehensive search of the literature was conducted in the following databases; PubMed (U.S. National Library of Medicine, USA), Web of Science [v.5.4] (Thomson Reuters, USA) and SciVerse Scopus (Elsevier Properties S.A, USA) for studies published before 31^st^ December 2011.

During the first stage the above databases were searched using the following search criteria. The PubMed database was searched using the MeSH (Medical Subject Headings) term diabetes mellitus and keywords Zinc, Zinc supplementation, Zn supplementation and Zinc therapy, Zn therapy. The Web of Science database was searched using search terms diabetes mellitus, Zinc supplementation, Zn supplementation and Zinc therapy Zn therapy in article topic. In the SciVerse Scopus database the search terms diabetes mellitus, Zinc supplementation, Zn supplementation and Zinc therapy Zn therapy in article title, abstract or keywords was used as the search criteria. Results were limited to studies on humans, published in English. conference proceedings, editorials, commentaries and book chapters/book reviews were excluded.

In the second stage the total hits obtained from searching the databases using the above search criteria was screened by reading the article title and abstracts. Studies not satisfying the inclusion criteria were excluded at this stage. In the third stage individual manuscripts were screened, and those not satisfying inclusion criteria were excluded. To obtain additional data a manual search of the reference lists of articles selected in stage three was performed. Wherever possible forward citations of the studies retrieved during the literature search was traced and screened for possible inclusion. This search process was conducted independently by two reviewers (PR and RJ) and the final group of articles to be included in the review was determined after an iterative consensus process.

### Data extraction and analysis

A meta-analysis of studies examining the effects of Zinc supplementation on the following clinical and biochemical parameters in patients with type-2 diabetes was performed (only 3 studies on type-1 diabetes); Fasting Blood Glucose (FBG), 2-h Post Prandial Blood Glucose (2-h PPBS), Glycated Haemoglobin (HbA1c), Total Cholesterol (TC), Low Density Lipoprotein Cholesterol (LDL-c), High Density Lipoprotein Cholesterol (HDL-c) and Triglycerides (TG). A fixed effect analysis was initially conducted for all comparisons. Heterogeneity was assessed using the ^2^ test on Cochranes *Q* statistic [25] and by calculating *I*^*2*^[26]. If significant heterogeneity was present (*p*<0.05 from ^2^ test) a random effects meta-analysis was carried out. Forest plots were used to illustrate the study findings and meta-analysis results. Data were analysed using RevMan version 5.1.2 (Review Manager, Copenhagen: The Nordic Cochrane Centre, The Cochrane Collaboration, 2011) statistical software package. In all analyses a *p*-value<0.05 was considered statistically significant.

FBG, 2-h PPBS, TC, LDL-c, HDL-c and TG are reported as mg/dl, where studies reported mmol/l a numerical conversion to mg/dl was done based on molecular weight. HbA1c is reported as a percentage. In studies using multiple supplements where possible the group receiving Zinc supplementation alone was compared with placebo group [27]. Where Zinc was supplemented in a single group together with other supplements (minerals and vitamins) we selected the group with the least number of additional supplements to compare with the placebo group [18,28-35]. In cross-over studies the pooled estimate of Zinc and placebo groups after completion of the entire cross-over scheme was used in the analysis [36,37].

## Results

### Literature search

**Figure 1:**
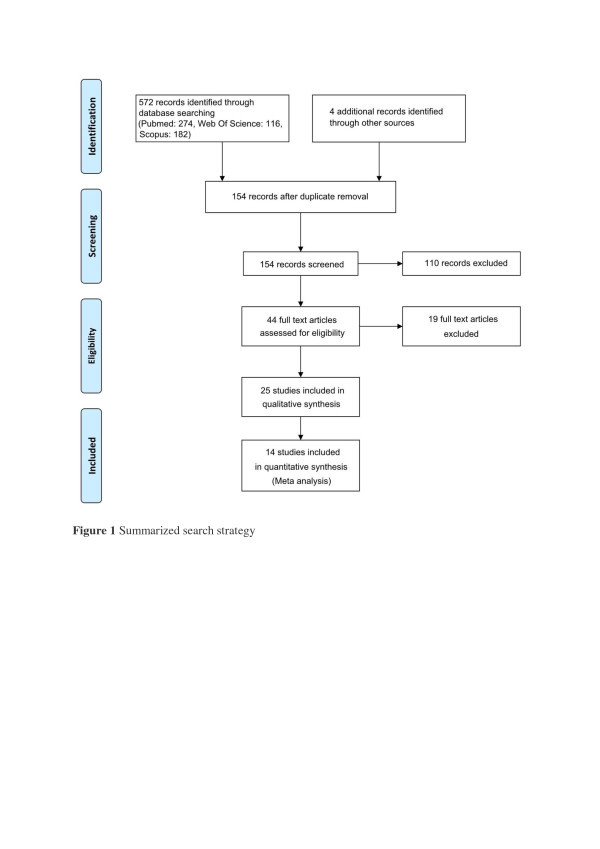
Summarized search strategy.

The literature search using the above search criteria identified the following number of articles in the respective databases; PubMed (n=274), Web of Science (n=116) and SciVerse Scopus (n=182). Four additional articles were identified by manually searching the reference lists and forward citations of included papers. After removing duplicates the total number of articles included in the present review is twenty five, which included 3 studies on type-1 diabetes and 22 studies on type-2 diabetes. The search strategy is summarized in Figure 1 and a description of the included studies provided in Table 1. The number of patients ranged from 13 to 110, and the duration of supplementation varied from 3week to 5years. There were nine studies where Zinc was used in a single group together with other vitamins and minerals [18,28-35], in all other studies Zinc was supplemented alone.

**Table 1:** Description of the included studies

Authors (reference), year, Country	Study design	Study description	Parameters studies	Formulation and Dose	Other tested substances	Significant outcomes
Afkhami-Ardekani et al., [19] 2008, Iran	R, DB, P*;*	T2DM (n=40); age 52.7 8.6year; 2 groups; 612weeks;	BMI; SBP; DBP; FBG; 2-h PPBS; HbA1c; TG; TC; HDL-c; LDL-c; Urea; Creatinine; ALT; AST	ZnSO_4_; 660mg/day^#^	None	Reduction in TG, TC, LDL and HbA1C
Al-Maroof et al., [20] 2006, Iraq	R, B, P;	T2DM (n=86); age 54.6 9.2year; 2 groups; 3months	HbA1c; FBG	ZnSO_4_; 30mg/day*	None	Reduction in HbA1C
Anderson et al., [27] 2001, Tunisia	R, DB, P;	T2DM (n=110); age 57.9 6.2year; 4 groups; 6months	Plasma and Urinary Zinc; Antioxidant effects	Zn gluconate; 30mg/day^#^	Chromium	Positive antioxidant effect
Blostein-Fujii et al., [38] 1997, USA	R, B, P;	T2DM (n=60); age 60.0 12.0year; 2 groups; 3weeks	Plasma Zinc; 5-nucleotidase; lipoprotein oxidation; IGF-1	30mg/day^#^	None	Raised IGF-1 (only if low at initiation)
De Sena et al., [39] 2005, Brazil	Casecontrol;	T1DM (n=37); age 4.1 16.5year; 2 groups; 4months	FBG; HbA1c; Urine sugar; Plasma and red cell Zn	7.5-15mg/day*	None	Increased erythrocyte Zinc concentration
Farvid et al., [28] 2011, Iran	R, DB, P;	T2DM (n=67); age 52.5 8.2year; 3 groups; 4months	Plasma Zinc; FBG; HbA1c; Fructosamine; Antioxidant effects; Neuropathy symptoms; Nerve conduction; Peripheral blood flow	ZnSO_4_; 20mg/day*	Mg^2+^; Vitamin B1,B2, B6,B12, C,E; Folate	Reduced severity of neuropathy symptoms in patients with mild-moderate neuropathy
Farvid et al., [29] 2005, Iran	R, DB, P;	T2DM (n=69); age 50.0 9.0year; 4 groups; 3months	Plasma and Urinary Zinc; SBP; DBP; MAP; FBG; HbA1c; TC; TG; HDL-c; LDL-c; Apo-A1; Apo B; Fructosamine; Urinary Microalbumin and protein	ZnSO_4_; 30mg/day*	Mg^2+^; Vitamin C,E	Lowered urinary albumin excretion
Farvid et al., [30] 2004a, Iran	R, DB, P;	T2DM (n=69); age 50.3 8.2year; 4 groups; 3months	Plasma and Urinary Zinc; SBP; DBP; MAP	ZnSO_4_; 30mg/day*	Mg^2+^; Vitamin C,E	Combination of vitamins and minerals decreases blood pressure
Farvid et al., [31] 2004b, Iran	R, DB, P;	T2DM (n=69); age 50.3 8.2year; 4 groups; 3months	Plasma and Urinary Zinc; TC; HDL-c; LDL-c; TG; Apo-A1; Apo B	ZnSO_4_; 30mg/day*	Mg^2+^; Vitamin C,E	co-supplementation of Mg, Zn, Vitamins C and E, increases HDL and Apo-A1
Faure et al., [17] 1995, France	Casecontrol;	T1DM (n=18); age 32.2 8.1year; 2 groups; 3months	Plasma Zinc; Antioxidant effects	Zn gluconate; 30mg/day*	None	Reduced lipid peroxidation
Garcia-Medina et al., [32] 2011, Spain	R; P;	T2DM (n=97); age 54.8 11.6year; 2 groups; 60months	Antioxidant effects; Visual acuity; Slit-lamp examination; IOP; Fundoscopy	27mg/day*	Cu^2+^, Mn^2+^, Se^2+^, Vitamin C, E, Niacin	Retardation of retinopathy progression; improved antioxidant status
Gunasekara et al., [33] 2011, Sri Lanka	R, B, P;	T2DM (n=96); age 53.4 7.2year; 3 groups; 4months	FBG; 2-h PPBS; HbA1c; Creatinine; Insulin; Plasma and Urinary Zinc; TC; TG; HDL-c; LDL-c	ZnSO_4_; 22mg/day*	Mg^2+^, Cu^2+^, Se^2+^, Multivitamin	Reduced HbA1C, FBG, 2-h PPBS, TC and TC/HDL ration.
Gupta et al., [40] 1998, India	R, DB, P;	T2DM (n=50); age 49.9 11.0year; 3 groups; 6weeks	Plasma Zinc; FBG; 2-h PPBS; Nerve conduction tests	ZnSO_4_; 660mg/day^#^	None	Reduced FBG and 2h-PPBS, improved motor nerve conduction velocity
Hayee et al., [41] 2005, Bangladesh	R, DB, P;	T2DM (n=60); age 50.9 11.1year; 3 groups; 6weeks	Plasma Zinc; FBG; 2-h PPBS; Nerve conduction tests	ZnSO_4_; 660mg/day^#^	None	Reduced FBG and 2h-PPBS, improved motor nerve conduction velocity
Hegazi et al., [42] 1992, Egypt	Casecontrol;	T2DM (n=56); age 26 62year; 2 groups; 3weeks	FBG; Plasma Zinc; Insulin; Glucagon; Glucose-6-Phosphatase	Zn_3_(PO_4_)_2_; 28mg/day^#^	None	Increased serum Insulin. Reduced FBG and Glucagon
Heidarian et al., [43] 2009, Iran	R, DB, C;	T2DM (n=50); age 53.2 9.2year; 2 groups; 3months	Plasma Zinc; Folate; Vitamin B12; Homocysteine	ZnSO_4_; 30mg/day^#^	None	Reduced serum Homocysteine, increase folate and Vitamin B12
Hussain et al., [34] 2006, Iraq	R, DB, P;	T2DM (n=46); age 49.1 6.0year; 3 groups; 3months	FBG; 2-h PPBS; HbA1c; C-Peptide	Zn acetate; 50mg/day^#^	Melatonin	Reduced FBG and 2-h PPBS
Kadhim et al., [35] 2006, Iraq	R, DB, P;	T2DM (n=46); age 49.1 6.0year; 3 groups; 3months	TC; TG; HDL-c; LDL-c; Creatinine; Urea; Urinary Microalbumin	Zn acetate; 50mg/day^#^	Melatonin	Reduced TC, TG and LDL
Niewoehner et al., [44] 1986, USA	Cohort study;	T2DM (n=13); age 61.0 2.0year; 2 groups; 68weeks	Plasma Zinc; HbA1c; Immune function	ZnSO_4_; 660mg/day^#^	None	No beneficial effects observed
Parham et al., [36] 2008, Iran	R, DB, C;	T2DM (n=39); age 53.2 9.2year; 2 groups; 6months	FBG; HbA1c; GFR; SBP; DBP; TC; TG; HDL-c; LDL-c; Urinary Albumin; Plasma Zinc	ZnSO_4_; 30mg/day*	None	Reduced urinary albumin excretion
Partida-Hernndez et al., [37] 2006, Mexico	R, DB, C;	T2DM (n=27); age 35 65year; 2 groups; 4months	FBG; HbA1c; Plasma Zinc; TG; TC; LDL-c; HDL-c	ZnSO_4_; 100mg/day^#^	None	Reduced TC and TG. Increased HDL
Raz et al., [45] 1989, Israel	Cohort study;	T2DM (n=13); age 55.0 2.4year; 1 group; 2months	Plasma Zinc; FBG; Fructosamine; HbA1c; TC; TG; HDL-c; immune function; IV GTT	ZnSO_4_; 660mg/day^#^	None	Increased glucose intolerance
Roussel et al., [46] 2003, Tunisia	R, DB, P;	T2DM (n=56); age 53.6 1.5year; 2 groups; 6months	Plasma and Urinary Zinc; Antioxidant effects	Zn gluconate 30mg/day*	None	Positive antioxidant effect
Seet et al., [47] 2011, Singapore	R, B, P;	T2DM (n=40); age 56.0 7.5year; 2 groups; 3months	FBG; Insulin; TC; LDL-c; HDL-c; TAG; PAF-AH; PLA_2_	Zn gluconate; 240mg/day^#^	None	No beneficial effects observed
Shidfar et al., [18] 2010, Iran	R, DB, P;	T1DM (n=48); age 720year; 2 groups; 3months	BMI; Apo A-1 and B; FBG; Insulin	ZnSO_4_; 10mg/day*	Vitamin A	Increase in serum Apo-A1 and Decrease in Apo-B/Apo-A1 ratio

ALT-Alanine Aminotransferase; AST-Aspartate Aminotransferase; BMI-Body Mass Index; B-Single blinded; C-Cross over; DB-Double Blinded; FBG-Fasting Blood Glucose; HbA1c-Glycated haemoglobin; HDL-c-High Density Lipoprotein-Cholesterol; IGF1-Insulin Like Growth Factor-1; IOP-Intra Ocular Pressure; IV GTT-Intravenous Glucose Tolerance Test; LDL-c-Low Density Lipoprotein-Cholesterol; MAP-Mean Arterial Pressure; P-Parallel; RRandomized; T1DM-Type 1 Diabetes; T2DM Type 2 Diabetes; TC-Total Cholesterol; TG-Triglycerides;

Age presented as meanSD in years where data were available and as age range in other studies; * - Elemental Zinc dose supplemented per day, # - Dosage given as formulations

### Effects on glycaemic control

**Figure 2:**
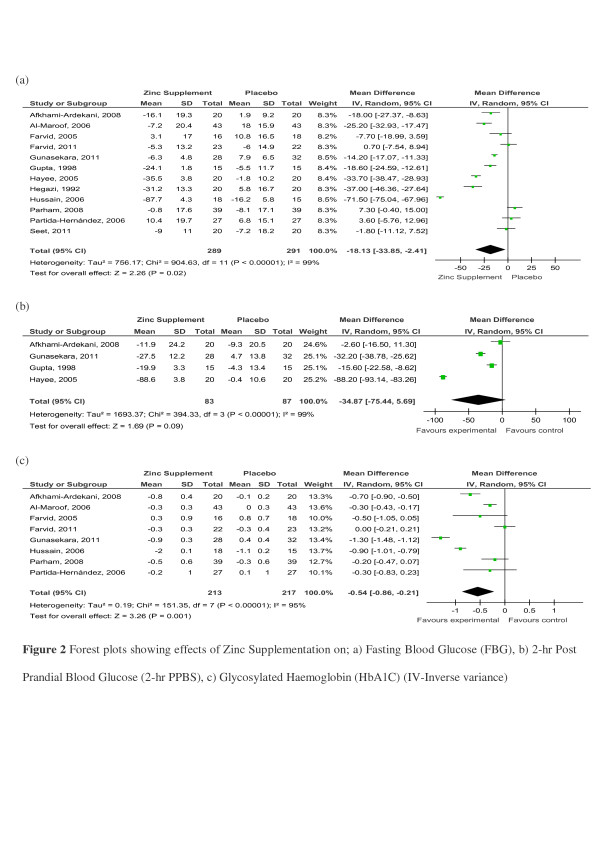
Forest plots showing effects of Zinc Supplementation on; a) Fasting Blood Glucose (FBG), b) 2-h Post Prandial Blood Glucose (2-h PPBS), c) Glycated Haemoglobin (HbA1C) (IV-Inverse variance).

The effects of Zinc supplementation on glycaemic control in patients with type-2 diabetes mellitus was evaluated by pooling data for FBG, 2-h PPBS and HbA1_C_. There were 12 studies comparing the effects of Zinc supplementation on FBG in patients with Type-2 Diabetes [19,20,28,29,33,34,36,37,40-42,47]. A forest plot of these studies is shown in Figure 2a. The pooled mean difference for FBG between Zinc supplemented and placebo groups from random effects analysis was 18.13mg/dl (95% CI:33.85,2.41; *p*<0.05). However statistical heterogeneity as indicated by I^2^=99% (*p*<0.001) of the data prevents the evaluation of a pooled estimate for FBG. The forest plot for 2-h PPBS (n=4) also shows a similar distinct reduction in (34.87mg/dl [95% CI:75.44; 5.69]) the Zinc treated group in comparison to controls (Figure 2b). The reduction in HbA1_C_ (n=8) was 0.54% (95% CI:0.86;0.21) in the Zinc treated group in comparison to controls (Figure 2c). However statistical heterogeneity as indicated by I^2^=95% (*p*<0.01) prevents further evaluation of a combined effect. Studies using Zinc supplement alone also demonstrated similar reductions in FBG, 2-h PPBS and HbA1c ( Additional file 1).

### Effects on lipids

**Figure 3:**
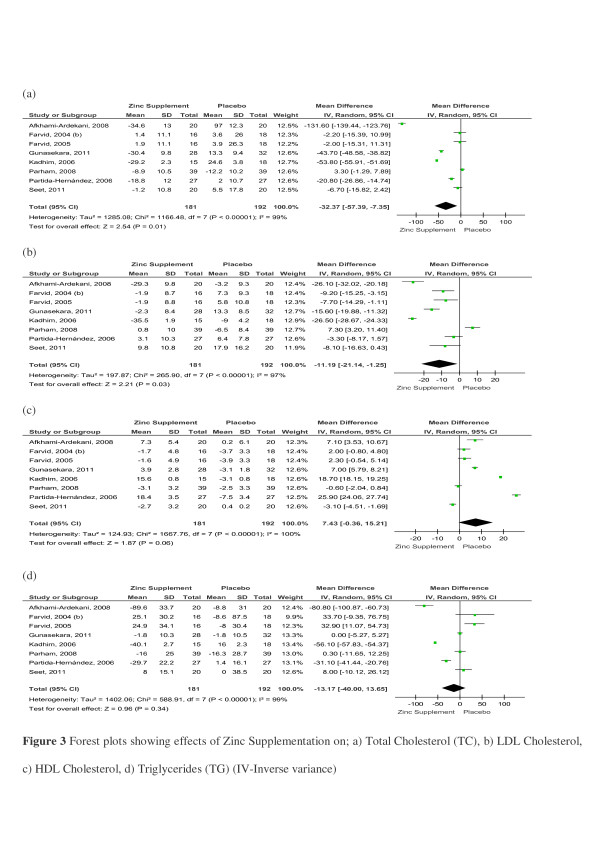
Forest plots showing effects of Zinc Supplementation on; a) Total Cholesterol (TC), b) LDL Cholesterol, c) HDL Cholesterol, d) Triglycerides (TG) (IV-Inverse variance).

The effects of Zinc supplementation on lipids in patients with type-2 diabetes mellitus was evaluated by pooling data for TC, LDL-c, HDL-c and TG. There were 8 studies comparing the effects of Zinc supplementation on each of the above lipid parameters in patient with Type-2 Diabetes [19,29,31,33,35-37,47]. The pooled mean difference for TC between Zinc supplemented and placebo groups from random effects analysis was 32.37mg/dl (95% CI:57.39,7.35; *p*<0.05). However statistical heterogeneity as indicated by I^2^99% (*p*<0.001) of the data prevents the evaluation of a pooled estimate for TC (Figure 3a). The forest plot for LDLc also shows a similar distinct reduction in the Zinc treated group in comparison to controls, the pooled mean difference from random effects analysis was11.19mg/dl (95% CI:21.14,1.25; *p*<0.05) (Figure 3b). The pooled mean difference for HDL-c showed an increase in the Zinc treated group in comparison to controls, however the overall effect was not significant (Figure 3c). Similarly the overall reduction observed in TG in Zinc supplemented groups in comparison to controls was not statistically significant (Figure 3d). Studies using Zinc supplement alone also demonstrated similar reductions in TC, LDL and TG, and an increase in HDL ( Additional file 2).

### Other significant effects

Studies have shown a significant reduction in systolic blood pressure after Zinc supplementation, the reduction varied from 2.4%6.1% [19,29,30,36]. Similarly a significant reduction in diastolic blood pressure (1.3%7.1%) has also being demonstrated in several studies [19,29,30,36]. Zinc supplementation has shown potential beneficial antioxidant effects in people with Type 2 Diabetes [27]. Increased levels of plasma Thio-Barbituric Acid Reactive Substances (TBARS) (a marker of lipid peroxidation) was observed in patients with both type-1 and type-2 diabetes [17,27,46]. Plasma TBARS i.e. lipid peroxidation decreased significantly after receiving Zn supplementation in both types of patients [17,27,46]. A study by Seet et al., using more reliable biomarkers of oxidative damage (F2-isoprostanes, neuroprostanes, cholesterol oxidation products and allantoin) in patients with type-2 diabetes with normal zinc levels contradicts this result and show that Zinc supplementation does not have any impact on oxidative damage and vascular function [47]. Antioxidant metalloenzyme activity of red cell Cu-Zn SOD (Super-Oxide Dismutase) was not significantly altered [27,46]. However, in type-1 diabetes patients with retinopathy Zinc supplementation significantly increased the activity of Cu-Zn SOD [17]. In patients with Type 2 Diabetes who had low levels of Insulin-like Growth Factor (IGF-1), Zinc supplementation significantly increased IGF-1 concentrations [38].

Studies evaluating the effects of micronutrients supplementation on various degrees and manifestations of diabetic neuropathy have shown that pharmacological treatment with vitamin C and E, Magnesium and Zinc supplementation ameliorate severity of neuropathy symptoms in diabetic patients with mild to moderate peripheral neuropathy [28]. Studies using Zinc supplementations alone have also demonstrated a significant improvement in motor nerve conduction velocity following supplementation in patients with type-2 diabetes [40,41]. However, autonomic functions have remained unchanged [40]. Similarly micronutrient supplementation including vitamins C and E together with Magnesium and Zinc significantly lowered urinary albumin excretion (a marker for glomerular dysfunction) in patients with type-2 diabetes [29]. In addition, Zinc supplementation alone also reduced albumin excretion in microalbuminuric patients with type-2 diabetes [36].

Hegazi, et al. reported that Zinc supplementation in patients with type-2 diabetes improved insulin secretions, while suppressing glucagon and glucose-6-phosphatase levels [42]. However, this effect on serum insulin has been contradicted by other studies [18,33,47]. Furthermore, Zinc supplementation has resulted in reduced serum homocysteine concentrations and increased vitamin B12 and folate concentrations in type 2 diabetic patients with microalbuminuria [43].

### Adverse effects

Afkhami-Ardekani et al., reported two cases of mild abdominal pain in patients receiving Zinc sulfate 660mg/day for 12weeks [19]. However, whether they were directly attributable to Zinc i.e. a cause-effect relationship has not been established. There have not been significant effects on renal and liver functions due to Zinc supplementation, as observed by unchanged blood urea, serum creatinine levels and plasma AST/ALT levels in those receiving Zn sulfate (22mg/day) and Zn acetate (50mg/day) for a period of 34months [33,35].

## Discussion

This is the first comprehensive systematic review and meta-analysis of studies evaluating the effects of oral Zinc supplementation in patients with diabetes mellitus. We summarize the data from 25 studies, involving a total of 1,362 patients. Although there is a considerable heterogeneity amongst the studies, our data shows several beneficial metabolic and clinical effects due to Zinc supplementations in patients with diabetes mellitus, namely improved glycaemic control and lipid parameters, with probable improvement in anti-oxidant status.

Glycaemic control is one of the most important therapeutic challenges in present day diabetes care; our meta-analysis shows that Zinc supplementation causes significant reduction in FBG, PPBG and HbA1c in patients with type-2 diabetes. Several molecular mechanisms are believed to be involved in the regulation of blood glucose levels following Zinc supplementation. *In-vitro* and *in-vivo* studies have demonstrated the insulin mimetic and hypoglycemic properties shown by Zinc (ii) complexes [48]. The protein tyrosine phosphatase 1B (PTP 1B), a key regulator of the phosphorylation state of the insulin receptor is known to be a target of Zinc ions [49]. Studies have shown that Zinc may play a role in improving peripheral insulin sensitivity, as it can potentiate insulin-stimulated glucose transport [50]. It also appears that Zinc has vital functional roles in cell physiology [51]. Recent genome wide association studies have found the islet-restricted Zinc transporter ZnT8 (SLC30A8) as a potential controller of insulin secretion and hence may modulate the risks of developing type 2 diabetes [52]. Our results show that post-supplementation HbA1c values were significantly reduced in the Zinc treated groups compared with controls. The pooled reduction of HbA1c was close to 0.6%, a magnitude that is clinically significant. In the UK Prospective Diabetes Study (UKPDS), patients randomized to intensive glycaemic control with Metformin reported a similar reduction of HbA1c by 0.6% compared to conventional treatment, which resulted in a 32% risk reduction for diabetes-related clinical end points and 42% for diabetes-related deaths [53]. However, as the magnitude of any fall in HbA1c is dependent upon several factors such as; baseline HbA1_C_, background therapy and endogenous -cell function [54], it is not advisable to directly compare the efficacy of Zinc with other glucose lowering agents without the availability of comparable clinical trial data.

Zinc supplementation resulted in a significant reduction of plasma total cholesterol, LDL-c and TAG, while increasing HDL-c levels in patients with type-2 diabetes, These findings are in contrast to results from a previous meta-analysis of controlled trials involving healthy subjects, where no beneficial effects of Zinc supplementation were observed on plasma total cholesterol, LDL-c, HDL-c or TAG concentrations [55]. The same meta-analysis showed that Zinc supplementation among healthy individuals was associated with a significant reduction of plasma HDL-c concentrations (7% decrease from baseline) [55]. In contrast to our findings, the above meta-analysis suggests that Zinc supplementation in healthy individuals may have detrimental health effects. This relationship between Zinc supplementation in healthy individuals and HDL-c levels appears to an extent to depend upon Zinc dosage and duration of supplementation [55]. Serum HDL-c levels appear to decrease at Zinc doses of over 50mg/day given for at least three months [56]. In addition to the lowering of HDL-c by zinc supplementation in health individuals, several adverse effects are also reported in the literature. A Randomized Controlled Trial (RCT) amongst an elderly group reported a significantly higher incidence of circulatory adverse effects in the Zinc supplemented group (80mg Zn/day), however the exact nature of these events were not reported [57]. Studies have shown that sustained hyperzincaemia may predispose individuals to thrombogenesis [56]. The health professionals follow-up study demonstrated an increased relatively risk of 2.37 fold for advanced prostate cancer in males who were supplemented with Zinc more than 100mg/day [58]. In addition, more than 150mg of Zinc per day may cause immune dysfunctions [59]. However, studies have shown that physiological doses of Zinc supplementation (20mg/day) for short durations (2months) produced favorable effects on nutritional and immune status in marginally Zinc deficient elderly individuals, while there were no significant changes observed in the plasma HDL-c levels [60]. Hence, from these findings we can postulate that supplementing Zinc may favorably alter lipid metabolism in patients with diabetes, but not in healthy individuals. Most of the studies included in the present meta-analysis were conducted in low-middle income developing countries where marginal Zinc deficiency might be highly prevalent, amongst patients with diabetes. Thus, it appears that the beneficial effects of Zinc supplementation on metabolic parameters can be seen mainly in individuals with Zinc deficiency or diseases causing Zinc deficiency such as diabetes.

Diabetes mellitus is characterized by hyperglycemia together with biochemical alterations of glucose and lipid metabolism. Furthermore diabetes results in increased oxidative stress, which also plays a major role in its pathogenesis [61]. In addition to the hypoglycemic and lipid lowering effects of regular Zinc supplementation in patients with diabetes, our results show that it reduces lipid peroxidation and hence demonstrate antioxidant effects [17,27,46]. Antioxidant properties of Zinc have long been recognized [62]. Eight weeks of Zinc supplementation in healthy volunteers showed reduction in plasma levels of lipid peroxidation products, DNA adducts and reduced mRNA for TNF- and IL-1 compared to control groups [63]. Oxidative stress and oxidative damage to tissues is known to occur in diabetes and may be associated with its complications. Farvid and coworkers [28,29] reported beneficial effects on diabetes neuropathy and nephropathy by Zinc supplementation. Thus it is possible to hypothesis that reduction in diabetes complication may be due to reduction of oxidative damage from Zinc supplementation. However, all these studies supplemented other antioxidant vitamins and minerals together with Zinc, hence it is difficult to conclude all these beneficial effects are due to Zinc supplements alone. The effect of Zinc on increasing IGF-1 concentration is mediated at molecular level by the Zinc/Growth Hormone-receptor complex responsible for IGF-1 mRNA synthesis, expression and stability [64]. The effect of supplemental Zinc on serum homocysteine concentration may be due to an influence on methionine synthase enzyme. Zinc is required for the binding of homocysteine to methionine synthase for its conversion to methionine [43]. Vitamin B12 is formed during this conversion, which in turn increases tetra-hydro folate production and hence the increase in Vitamin B12 and folate following Zinc supplementation.

We acknowledge several limitation to the extent to which conclusions can be drawn from the present systematic review. There is a considerable heterogeneity amongst the included studies, which stems from; a) Variations in baseline parameters such as serum Zinc status, blood glucose and lipid levels, b) Differences in Zinc doses, formulae, sample sizes and study durations, and c) Limited availability of data on Zinc intake from other sources such as diet. In addition several studies have supplemented Zinc together with other vitamins and minerals in a single group, and these vitamins and minerals could have acted as potential confounding factors masking/enhancing the isolated effects of Zinc supplementation on diabetes. However, results from studies using Zinc supplementation alone also show more or less similar findings and a study that supplemented with Zinc+MVM (Multi Vitamin Mineral) and MVM without Zinc reported that only patients with diabetes receiving Zinc+MVM showed beneficial metabolic effects [33]. Hence, it is reasonable to postulate that these effects could be the result of Zinc supplementation. Furthermore, studies using Zinc supplement alone also demonstrated similar beneficial effects on glycaemic control and lipid parameters (Additional file 1 and 2).

In conclusion, this first comprehensive systematic review and meta-analysis on the effects of Zinc supplementation in patients with diabetes demonstrates that Zinc supplementation has beneficial effects on glycaemic control and promotes healthy lipid parameters. However, individual studies showed considerable heterogeneity. Further studies are required to identify the exact biological mechanisms responsible for these results. In addition, at present there is only one short-term (4weeks) study investigating the effects of Zinc supplementation in pre-diabetes [65]. Hence it is also important to conduct further well design randomized control trials in those with pre-diabetes to evaluate potential beneficial effects of Zinc supplementation in prevention of diabetes.

## Abbreviations

FBG = Fasting Blood Glucose; HbA1c = Glycated hemoglobin; HDL-c = High Density Lipoprotein Cholesterol; LDL-c = Low Density Lipoprotein Cholesterol; TG = Triglycerides; TC = Total Cholesterol; RCT = Randomized Control Trial; MVM = Multi Vitamin Mineral.

## Competing interests

The authors declare that they have no competing interests

## Authors' contributions

RJ, PR, PG, RLDKM, GRC and PK made substantial contribution to conception and study design. RJ and PR were involved in data collection. RJ, PR, RLDKM, and PK were involved in refining the study design, statistical analysis and drafting the manuscript. PG, PK and GRC critically revised the manuscript. All authors read and approved the final manuscript.

## Supplementary Material

Additional file 1 Forest plots showing effects of Zinc Supplementation alone on; a) Fasting Blood Glucose (FBG), b) 2-hr Post Prandial Blood Glucose (2-hr PPBS), c) Glycosylated Haemoglobin (HbA1C) (IV-Inverse variance) Click here for file

Additional file 2 Forest plots showing effects of Zinc Supplementation alone on; a) Total Cholesterol (TC), b) LDL Cholesterol, c) HDL Cholesterol, d) Triglycerides (TG) (IV-Inverse variance) Click here for file
