# Supplementary material for: Effects of zinc supplementation on diabetes mellitus: a systematic review and meta-analysis
Source: Diabetol Metab Syndr. 2012 Apr 19;4:13. doi: 10.1186/1758-5996-4-13 (PMC3407731; doi:10.1186/1758-5996-4-13)
Supplement: Additional file 1 — Forest plots showing effects of Zinc Supplementation alone on; a) Fasting Blood Glucose (FBG), b) 2-hr Post Prandial Blood Glucose (2-hr PPBS), c) Glycosylated Haemoglobin (HbA1C) (IV-Inverse variance) [file 1758-5996-4-13-S1.doc]

a)

b)

c)

Forest plots showing effects of Zinc Supplementation alone on; a) Fasting Blood Glucose (FBG), b) 2-hr Post Prandial Blood Glucose (2-hr PPBS), c) Glycosylated Haemoglobin (HbA1C) (IV-Inverse variance)
