# Supplementary material for: Effects of zinc supplementation on diabetes mellitus: a systematic review and meta-analysis
Source: Diabetol Metab Syndr. 2012 Apr 19;4:13. doi: 10.1186/1758-5996-4-13 (PMC3407731; doi:10.1186/1758-5996-4-13)
Supplement: Additional file 2 — Forest plots showing effects of Zinc Supplementation alone on; a) Total Cholesterol (TC), b) LDL Cholesterol, c) HDL Cholesterol, d) Triglycerides (TG) (IV-Inverse variance) [file 1758-5996-4-13-S2.doc]

a)

b)

c)

d)

Forest plots showing effects of Zinc Supplementation on; a) Total Cholesterol (TC), b) LDL Cholesterol, c) HDL Cholesterol, d) Triglycerides (TG) (IV-Inverse variance)
